# Draft Whole-Genome Sequences of the Polar Cyanobacterium *Leptolyngbya* sp. Strain Cla-17 and Its Associated Flavobacterium

**DOI:** 10.1128/mra.00059-22

**Published:** 2022-06-27

**Authors:** Bérangère Péquin, Julien Tremblay, Christine Maynard, Jessica Wasserscheid, Charles W. Greer

**Affiliations:** a McGill University, Department of Natural Resource Sciences, Ste-Anne-de-Bellevue, Quebec, Canada; b Energy, Mining, and Environment Research Centre, National Research Council Canada, Montreal, Quebec, Canada; University of Southern California

## Abstract

Draft whole-genome sequences of a coculture are presented. One component was a polar cyanobacterium, *Leptolyngbya* sp. strain Cla-17. The second was a heterotrophic bacterium, Flavobacterium saccharophilum, found in the phycosphere of the cyanobacterium.

## ANNOUNCEMENT

Cyanobacteria and some heterotrophic bacteria interact closely in the phycosphere microenvironment (1), which is conducive to various molecular exchanges between species (2). Because of these relationships, many cyanobacteria cannot grow in axenic cultures (3). Here, we report the draft whole-genome sequences of an Arctic cyanobacterium and its associated flavobacterium.

The cyanobacterium Cla-17 (strain PCCC_Cla17 from the Polar Cyanobacteria Culture Collection [PCCC]) was cultured from Char Lake snow (74°42′30″N, 94°53′0″W) in 2008 by Harding and coworkers (4). Briefly, snow was melted and filtered through 0.2-μm polycarbonate filters, and then the filters were incubated in liquid BG-11 medium (5). The culture was first exposed to natural light from Resolute Bay at 16°C; once back in the university laboratory, it was cultivated at 10°C under continuous light at 50 μmol photons m^−2^ s^−1^. The culture was kindly provided by Professor W. Vincent (Centre d’Études Nordiques [CEN], Université Laval, Québec, Canada). For sequencing, the culture was grown on liquid BG-11 medium at 14°C with a 12-h/12-h light/dark cycle at 5 to 28 μE m^−2^ s^−1^ irradiation for 5 weeks. As reported here, this cyanobacterium grows in a mixed culture with a heterotrophic flavobacterium.

DNA was extracted using the DNeasy UltraClean microbial kit (Qiagen). A short-read library was prepared using the QIAseq FX DNA library kit (Qiagen) and sequenced on a MiSeq system (Illumina) using v2 chemistry (2 × 250 bp). A 20-kb SMRTbell library was prepared and sequenced with one single-molecule real-time (SMRT) Cell on a Sequel system (Pacific Biosciences [PacBio]) using v3.0 chemistry at the Génome Québec Innovation Center (McGill University, Montréal, Canada). Genomic analyses were performed with the default settings for all software unless otherwise noted.

The *de novo* assembly was carried out using the Hierarchical Genome Assembly Process (HGAP4) (6) in SMRT Link v6.0.0. The coverage cutoff value was set to 30× and the estimated genome size to 5 million bp. Raw subreads (1,210,976 subreads) with a read quality (RQ) value of <0.7 (pbcoretools v1.5.0) were omitted, and the remaining subreads (151,243 subreads) were used as input for the FALCON assembler (falcon-kit v1.2.2) (7). Polishing of the assembly was performed with Arrow v2.2.2 in SMRT Link v6.0.0 to give a total of 24 contigs, representing 12,039,402 bp. The polished assembly was scaffolded using SSPACE-LongRead (8) with default parameters and gave 10 scaffolds, totaling 12,173,746 bp. The presence/absence of circularity of the genomes and the overlapping ends were assessed and, if necessary, removed in the postprocessing steps of the SSPACE-LongRead. The genomes were not rotated to a certain base.

To further correct for artifacts, MiSeq sequencing data generated with the same starting DNA were aligned against scaffolds (BWA v0.7.17) (9). Ten consensus scaffolds were generated with bcftools v1.9 (10), more precisely with (i) bcftools mpileup, (ii) bcftools call, and (iii) bcftools norm. Plasmids were identified with PlasFlow v1.1 (11) and were not circularized. Final corrected scaffolds were annotated using the NCBI Prokaryotic Genome Annotation Pipeline (PGAP) (12). The genomes were estimated to be highly complete by CheckM v1.0.4 (13). Finally, the taxonomic assignment was performed with CAT v5.2.3 (14). The results showed two very different bacterial species that are phylogenetically distant (*Flavobacterium* versus *Leptolyngbya*). The genome statistics for each strain are indicated in Table 1.

**TABLE 1:** Genome statistics for each strain

Parameter	Data for:	
	*Leptolyngbya* sp.	Flavobacterium saccharophilum
No. of reads		
Illumina	2,640,000	
PacBio	151,471	
PacBio raw read *N*_50_ (bp)	10,231	
Total genome size (bp)	6,647,508	5,526,236
Size of putative chromosomal contigs (bp)	Contig 1: 2,498,688; contig 2: 2,920,439	Circular contig: 5,526,236
Size of other types of contigs (bp)	Contig 3: 94,057	None
Total no. and size (bp) of plasmids	6, ranging from 49,058 to 480,286	None
Chromosome *N*_50_ (bp)	2,498,684	5,526,236
Coverage (×)		
Illumina	108	108
PacBio	400	400
GC content (%)	Contig 1: 46.81; contig 2: 47.34	35.75
No. of genes	6,226	4,570
No. of rRNAs	3	5
No. of tRNAs	204	56
CheckM completeness (%)	96.62	99,65
CheckM contamination (%)	0.12	0.71
Accession no.		
Assembly	GCA_016807185.1	GCA_013112255.2
BioSample	SAMN17309407	SAMN14367226

It is known that *Flavobacterium* species are regularly found with cyanobacteria. They can inhibit or enhance the growth of cyanobacteria and degrade compounds synthesized by cyanobacteria (15). However, the ecological implications of these close interactions remain largely unknown.

### Data availability.

This whole-genome shotgun project has been deposited in GenBank under the accession number JAEVYN000000000. Raw reads are available under the BioProject accession number PRJNA612312. The SRA accession numbers for the raw PacBio Sequel and MiSeq data are SRR11301546 and SRR11301545, respectively.
